# Identification of *Leishmania* Species in Naturally Infected Sand Flies from Refugee Camps, Greece

**DOI:** 10.3201/eid2502.181359

**Published:** 2019-02

**Authors:** Emmanouil A. Fotakis, Ioannis A. Giantsis, Aimilia Avgerinou, Sofoklis Kourtidis, Evangelia Agathaggelidou, Christina Kapoula, Glykeria Dadakou, John Vontas, Alexandra Chaskopoulou

**Affiliations:** Agricultural University of Athens, Athens, Greece (E.A. Fotakis, J. Vontas);; Foundation for Research and Technology Hellas, Heraklion, Greece (E.A. Fotakis, J. Vontas);; US Department of Agriculture, Agricultural Research Service—European Biological Control Laboratory, Thessaloniki, Greece (I.A. Giantsis, A. Avgerinou, A. Chaskopoulou);; General Directorate of Public Health and Social Welfare, Region of Central Macedonia, Thessaloniki (S. Kourtidis, E. Agathaggelidou, C. Kapoula, G. Dadakou)

**Keywords:** *Leishamania tropica*, *Leishmania donovani*, sand flies, refugee camps, parasites, Greece

## Abstract

High infection rates of *Leishmania donovani* and *L. tropica* were detected in *Phlebotomus* spp. sand flies collected from refugee camps in Greece, indicating increased risk of infection among local populations. Detection and treatment of leishmaniasis, community education, and vector control are essential measures to prevent pathogen transmission and protect public health.

Leishmaniasis is a parasitic disease caused by protozoa of the genus *Leishmania*, which are transmitted by sand flies of the genus *Phlebotomus*. Visceral leishmaniasis (VL) and cutaneous leishmaniasis (CL) are endemic in southern Europe; *L. infantum* is the primary causative agent (*1*). An emerging problem regarding leishmaniasis control in Europe is the potential introduction of new *Leishmania* species, such as *L. donovani* and *L. tropica*, through travelers, refugees, and immigrants from countries where these species are endemic. *L. tropica,* which has a limited presence in Europe and is reported mostly in Greece, causes anthroponotic CL; *L. donovani,* recently reported in Cyprus, causes anthroponotic VL and CL (*2*).

More than 1 million refugees and immigrants arrived in Greece in 2015 and 2016 (*3*), mostly from Syria, Iraq, and Afghanistan, where leishmaniasis poses a serious economic and social burden (*4*). Most of these persons are hosted in temporary accommodation sites (camps) throughout Greece. A vectorborne pathogen surveillance network targeting refugee camps was deployed in Greece during June–September 2017. Here we report major findings related to sand fly activity and *Leishmania* transmission associated with these temporary settlements.

## The Study

We surveyed 4 refugee camps in Greece in 2017: the Lagadikia and Diavata camps in the Thessaloniki Regional Unit, northern Greece, and the Vial and Souda camps on the island of Chios in the Northeastern Aegean Islands complex. Sand flies were collected from camps every 2 weeks from June through September by using CDC light traps (John W. Hock, Gainesville, FL, USA) baited with dry ice. For this analysis, we further included sand flies collected from the metropolitan area of Thessaloniki during 2011–2015 (*5*). 

Wild-caught sand flies were stored in ethanol. The head from each female sand fly was dissected from the body and stored individually to be used for sand fly species identification using a multiplex diagnostic assay based on species-specific single-nucleotide polymorphisms of the nuclear 18S rRNA gene (*6*); positive controls were previously identified sand flies (*6*). Sand fly heads were also used for *Leishmania* detection. The remaining parts of the abdomen and thorax from each female sand fly were pooled based on species, sampling site (refugee camp), and collection date (6–16 female specimens per pool). We extracted DNA from the pooled samples and individual sand fly heads using the NucleoSpin Tissue kit (Macherey-Nagel, Düren, Germany) according to the manufacturer’s instructions.

We screened DNA extracted from pools for the presence of *Leishmania* by amplification of a 300–350-bp fragment of the *Leishmania* DNA ribosomal internal transcribed spacer 1 (ITS1), as described by Rêgo et al. (*7*). We used *Phlebotomus argentipes* pools originating from laboratory colonies infected with *L. donovani* or *L. infantum* as positive controls and used male sand flies as negative controls. We analyzed amplicons by electrophoresis on a 2% agarose gel.

We individually screened the extracted DNA from the female heads corresponding to the positive pools for the presence of *Leishmania* as described, followed by *Leishmania* species identification applying the restriction fragment length polymorphism diagnostic test of Schönian et al. (*8*). *Leishmania* species were further confirmed in a subset of samples using ITS1 product sequencing and BLAST analysis (https://www.ncbi.nlm.nih.gov/BLAST*)*. We depicted the phylogenetic relationships of the ITS1 sequences with conspecific haplotypes obtained from the GenBank database by constructing a neighbor-joining dendrogram using MEGA version 6.05 (*9*) using pairwise gap deletion and performing 1,000 bootstrap replicates.

For discriminating between members of the *L. donovani* complex, we analyzed the cysteine protease b (*cpb*) gene, applying the species-specific PCR assay described by Hide and Bañuls (*10*). We amplified a segment of Heat-shock protein 70 (Hsp70) as described by Van der Auwera et al. (*11*) and then sequenced the amplicons.

**Figure 1:**
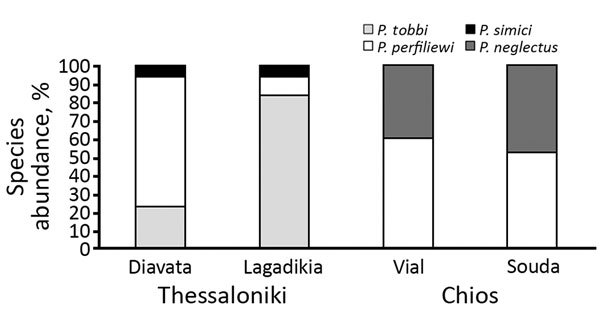
*Phlebotomus* spp. sand fly species composition and relative species abundance in Thessaloniki and Chios refugee camps, Greece.

We analyzed 10 sand fly pools (n = 127 individuals) from Thessaloniki refugee camps, 240 pools (n = 1200) from the Thessaloniki metropolitan region, and 5 pools (n = 61) from Chios refugee camps, comprising 6 different species (Figure 1; *5*). ITS1 PCR detected *Leishmania* DNA in 10 pools, all of which originated from the Thessaloniki refugee camps (Table 1). Of the 127 sand fly heads that were analyzed individually from the positive pools, we detected *Leishmania* in 26 sand flies from Lagadikia and 35 sand flies from Diavata (Table 2), corresponding to a natural *Leishmania* infection frequency of 43% for Lagadikia and 52% for Diavata. We detected *Leishmania* parasites in the dissected heads of all 3 prevalent sand fly species (*P. perfiliewi, P. tobbi, P. simici*), indicating late-stage transmissible infection. The absence of parasite detection from the broader region of Thessaloniki, in parallel with the unusually high infection frequencies observed in both Thessaloniki refugee camps, strongly indicates high levels of focal parasite transmission (*12*) in the Thessaloniki refugee camps.

**Table 1:** Sand fly pool grouping of refugee camp samples and *Leishmania* detection at pool level, Greece

Refugee camp	Sand fly species	No. specimens	No. pools	No. (%) *Leishmania-* positive pools
Lagadikia	*P. tobbi*	45	3	3 (100.0)
	*P. perfiliewi*	9	1	1 (100.0)
	*P. simici*	6	1	1 (100.0)
	Total	60	5	5 (100.0)
Diavata	*P. tobbi*	15	1	1 (100.0)
	*P. perfiliewi*	45	3	3 (100.0)
	*P. simici*	7	1	1 (100.0)
	Total	67	5	5 (100.0)
Souda	*P. perfiliewi*	11	1	0
	*P. neglectus*	10	1	0
	Total	21	2	0
Vial	*P. perfiliewi*	24	2	0
	*P. neglectus*	16	1	0
	Total	40	3	0

**Table 2:** *Leishmania* detection and species identification in individual sand flies in refugee camps, Greece

Refugee camp	Sand fly species	No.*	*Leishmania* infection			
			*L. donovani*†	*L. tropica*‡	Total§	Infection rate, %¶
Lagadikia	*P. tobbi*	45	14	7	21	43
	*P. perfiliewi*	9	3	1	4	
	*P. simici*	6	0	1	1	
Diavata	*P. tobbi*	15	1	2	3	52
	*P. perfiliewi*	45	21	9	30	
	*P. simici*	7	1	1	2	

*Total number of specimens tested per sand fly species per camp. †Total number of sand flies infected with *L. donovani* per sand fly species, per camp. ‡Total number of sand flies infected with *L. tropica* per sand fly species, per camp. §Total number of sand flies infected with *L. donovani* or *L. tropica* per sand fly species, per camp. ¶Total *Leishmania* infection rate per refugee camp.

*Leishmania* species identification through restriction fragment length polymorphism assays revealed 2 different restriction profiles, demonstrating the presence of *L. donovani* complex in 40 sand flies and *L. tropica* in 21 sand flies (Table 2). Sequencing results in a subset of the positive samples confirmed the identity of the *Leishmania* species. Two ITS1 haplotypes were found corresponding to each species and were deposited into GenBank (accession nos. MH763642 for *L. donovani* and MH763643 for *L. tropica*). Both cpb and Hsp70 gene analyses confirmed the identity of all 40 *L. donovani* complex isolates as *L. donovani donovani*. The cpb species–specific PCR assay amplified the 741-bp product that characterizes *L. donovani* (*10*). Hsp70 analysis resulted in a single haplotype (GenBank accession no. MH788969), 541 bp in length, that showed 100% sequence similarity with the Hsp70 reference sequences for *L. donovani* (*11*).

**Figure 2:**
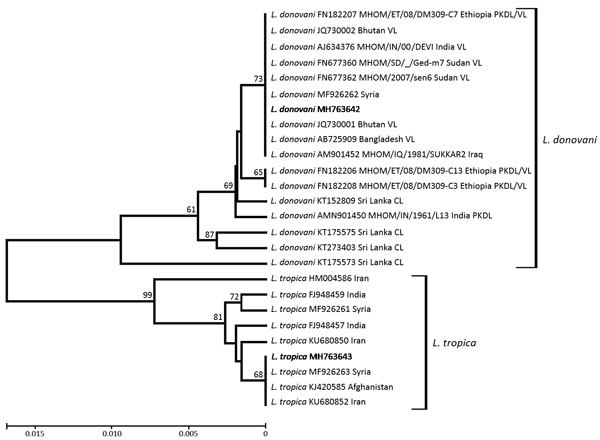
Dendrogram depicting internal transcribed spacer 1 genetic relationships of the 2 *Leishmania* haplotypes (bold) found in study of *Leishmania* spp. in naturally infected sand flies in refugee camps, Greece, compared with conspecific *Leishmania* haplotypes obtained from GenBank. CIs >60% are indicated next to the branches. Each taxon is identified by species name, GenBank accession number, World Health Organization code if available, country, and type of disease caused, if known. Scale bar indicates nucleotide substitutions per site. CL, cutaneous leishmaniasis; VL, visceral leishmaniasis.

Phylogenetic analysis demonstrated clustering of the detected *L. donovani* haplotype with conspecific haplotypes, mainly from Syria, Iraq, India, Sudan, and Ethiopia (Figure 2), that have been associated with VL in humans. The *L. tropica* haplotype was clustered with haplotypes from Syria, Afghanistan, and Iran.

## Conclusions

The leishmaniasis disease status of the refugee populations hosted in the temporary accommodation sites in Thessaloniki or elsewhere in Greece and Europe remains unknown. *L. donovani* is one of the main causative agents of anthroponotic VL, a dangerous form of leishmaniasis that is lethal if left untreated. CL is more benign, but lesions caused by *L. tropica* are generally more difficult to treat with antimonial drugs because of the development of drug resistance (*13*). An epidemic of CL was recently recorded in camps housing refugees from Syria within Lebanon’s borders; 85% of the reported cases were caused by *L. tropica* (*14*). The high *L. donovani* and *L. tropica* infection rates detected in natural sand flies from the refugee camps in northern Greece suggest that the persons accommodated in these settings face an increased risk for infection. It is therefore imperative to take all necessary precautions to prevent transmission within refugee populations, as well as in the surrounding communities.

Systematic active and passive detection of leishmaniasis within the refugee populations, effective treatment of infected patients, access to adequate living conditions, health education of the community, and establishment of targeted vector control activities are essential steps necessary to protect public health, as well as to avert the colonization of the local sand fly vectors by exotic *Leishmania* species. Studies investigating the initial *Leishmania* disease burden in refugee and immigrant populations when entering Europe and risk factors associated with disease transmission within the camp settlements are also required for efficient disease control.
